# Surface Modified Activated Carbons: Sustainable Bio-Based Materials for Environmental Remediation

**DOI:** 10.3390/nano11113140

**Published:** 2021-11-21

**Authors:** Manoj Kumar Jha, Sahira Joshi, Ram Kumar Sharma, Allison A Kim, Bishweshwar Pant, Mira Park, Hem Raj Pant

**Affiliations:** 1 Nanomaterial Lab, Department of Applied Sciences and Chemical Engineering, IOE, Tribhuvan Universtiy, Kathmandu, Lalitpur 44700, Nepal; jhamanoj144@gmail.com (M.K.J.); sjoshi61@hotmail.com (S.J.); rksharma2002@ioe.edu.np (R.K.S.); 2 Department of Healthcare Management, Woosong University, Daejeon 34606, Korea; allisonkim@wsu.ac.kr; 3 Carbon Composite Energy Nanomaterials Research Center, Woosuk University, Wanju 55338, Korea; 4 Woosuk Institute of Smart Convergence Life Care (WSCLC), Woosuk University, Wanju 55338, Korea

**Keywords:** agricultural waste, biomass, activated carbon, environmental remediation

## Abstract

Global warming and water/air contamination caused by human activities are major challenges in environmental pollution and climate change. The improper discharge of a large amount of agro-forest byproduct is accelerating these issues mainly in developing countries. The burning of agricultural byproducts causes global warming, whereas their improper waste management causes water/air pollution. The conversion of these waste materials into effective smart materials can be considered as a promising strategy in waste management and environmental remediation. Over the past decades, activated carbons (ACs) have been prepared from various agricultural wastes and extensively used as adsorbents. The adsorption capacity of ACs is linked to a well-developed porous structure, large specific surface area, and rich surface functional moieties. Activated carbon needs to increase their adsorption capacity, especially for specific adsorbates, making them suitable for specific applications, and this is possible by surface modifications of their surface chemistry. The modifications of surface chemistry involve the introduction of surface functional groups which can be carried out by various methods such as acid treatment, alkaline treatment, impregnation, ozone treatment, plasma treatment, and so on. Depending on the treatment methods, surface modification mainly affects surface chemistry. In this review, we summarized several modification methods for agricultural-waste-based ACs. In addition, the applications of AC for the adsorption of various pollutants are highlighted.

## 1. Introduction

Agricultural wastes have become a global issue in recent years due to inefficient disposal and poor management techniques mainly in developing countries. The majority of agricultural biomass residues are discarded to decompose naturally or are burned openly, which can generate harmful gases, smoke, and dust, thereby resulting in air, soil, and water pollution [1]. Therefore, it is important to address this environmental problem caused by agricultural byproducts (wastes). Utilization of agricultural wastes or biomasses for solving environmental problems could be considered as an effective and green strategy in environmental protection as they are inexpensive, renewable, and locally available in large quantities [2,3,4]. In the literature, agricultural wastes have been proposed as an economic and eco-friendly precursor for ACs [5,6,7,8,9]. The agricultural wastes are rich in lignocelluloses, mainly composed of polysaccharides such as cellulose (35–50%), hemicellulose (20–35%), and an aromatic polymer, lignin (10–25%) [10]. Hemicellulose and lignin act as matrix and encrusting ingredients, respectively, and surround cellulose to generate a skeleton [11]. Being lignocellulosic materials, they have unique chemical compositions with a low content of inorganic materials and a relatively high content of volatile organic materials. Such chemical composition allows them to synthesize ACs with a well-defined pore structure/network and extended surface area. This conversion could solve problems related to undesirable agricultural wastes by turning them into a useful, valuable adsorbent that can address problems associated with environmental pollution and waste management. The facile conversion of pollutant materials into useful ones that can be applied as an adsorbent to remove other pollutants present in the water/air becomes a blessing for material scientists and environmental engineers. Additionally, AC derived from lignocellulosic biomass wastes has the potential to be a low-cost substitute for non-renewable coal-based granular ACs, presuming they have equal or greater adsorption efficiency [12,13]. The biochemical compositions of some agriculture lignocellulosic biomasses are given in Table 1.

**Figure 1:**
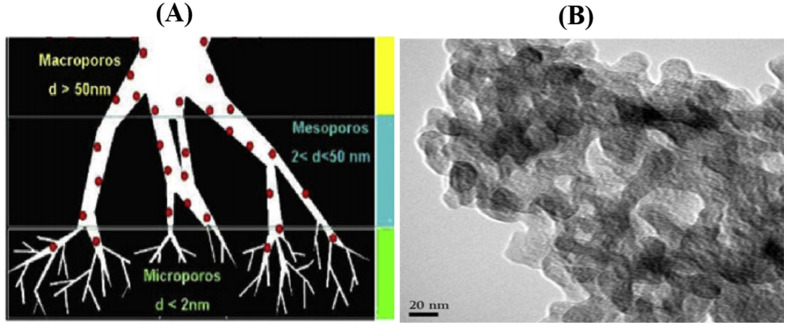
(**A**) Schematic diagram representing the three types of pores for pollutant adsorption [16]. Reprinted with permission from the *Journal of Analytical and Applied Pyrolysis*, Copyright Elsevier 2021. (**B**) TEM image of AC showing the hierarchical pore distribution [17]. Reprinted with permission from *Applied Surface Science*, Copyright Elsevier 2019.

AC is a versatile adsorbent having a complex pore structure, a large specific surface area, exceptional chemical stability, and extended functionalities on the outward [15]. These exceptional features have made it widely used in various applications such as water purification, gas separation and storage, catalysis, and supercapacitors. ACs can be synthesized from bio-wastes by a two-stage approach: (i) carbonization of a carbon-rich precursor and (ii) activation of the carbonized product. ACs can be prepared from the carbon-rich precursor by using thermal or chemical methods. By suitable thermal or chemical treatment, the non-carbon and volatile carbon species are removed, thereby leaving behind a fixed carbon mass with a primitive pore structure. The activation process involves exposing the ACs to an oxidizing agent at a high temperature, which forms new pores and vessels. Pores in ACs are usually categorized into three groups; (i) micropores (diameter less than 2 nm), mesopores (diameter ranging from 2 to 50 nm), and macropores (diameter greater than 50 nm) as shown in Figure 1A. The TEM image shown in Figure 1B also shows the hierarchical pore distribution of AC.

Another important characteristic of AC is the chemical composition of its surface. Heterogeneity of the chemical surface is crucial to the surface composition of the AC. The heteroatoms are generally oxygen, nitrogen, halogen, and other elements that are linked to the carbon layers’ edges. These heteroatoms constitute functional groups at the surface of ACs which consequently affect the hydrophilicity and hydrophobicity of the ACs [18]. Since the edges are the prime adsorbing surface, the presence of these heteroatoms at the edges highly influences the adsorptivity of the ACs. Hence, by modifying the morphological features along with the generation of diverse surface functionalities, the performance of ACs can be enhanced.

The functional moieties at the surface determine several characteristics of ACs such as wettability, surface charge, the electron density of the graphitic layers, and catalytic performance. These characteristics make the ACs suitable for various applications such as sensors [19], energy storage [20], catalytic reactions [21], and adsorptions [22,23]. The thermal or chemical post-treatments can change the nature and application of superficial groups [24]. A modified surface functional group on the surface of ACs enhances its adsorption capacity. Carboxyl, carbonyl, phenols, lactones, and quinines are some key functional groups that are helpful for pollutant adsorption. Modification of porous carbons’ surface chemistry (the process of customizing the surface of the AC) might be a promising path toward the specific application of these materials [25]. To regulate the hydrophilic/hydrophobic characteristics of ACs and increase their binding capacity with metals (or metal oxide), physical or chemical techniques are used to alter the superficial effective group on ACs and the ions and molecules put on the surface, making it the active site in particular adsorption. A modified AC with various functional groups can be applied for extracting metallic cations from any kind of solutions, catalysis, waste and hazardous effluent treatment, and so on. Some methods for changing the chemical composition of AC’s surface include surface oxidation, surface reduction, load material alteration, microwave processing modification, acid/alkali treatment, and grafting of different moieties [26,27,28]. The surface modification of ACs is carried out after the activation process. There are three different sorts of changes that may be done, viz. physical modification, chemical modification, and biological modification. In addition, oxidative and non-oxidative treatments have also been reported in the literature [29,30]. In the chemical process, acid, alkali, or salt are used to improve the surface functionalities, whereas the physical process is carried out to improve the physical characteristics. Moreover, the surface modification of ACs can be achieved by the combination of mechano-chemical and thermo-chemical processes. In addition, different functional moieties and metal/metal oxide NPs can be also grafted on the surface of AC for its surface modification [31,32,33,34,35].

**Figure 2:**
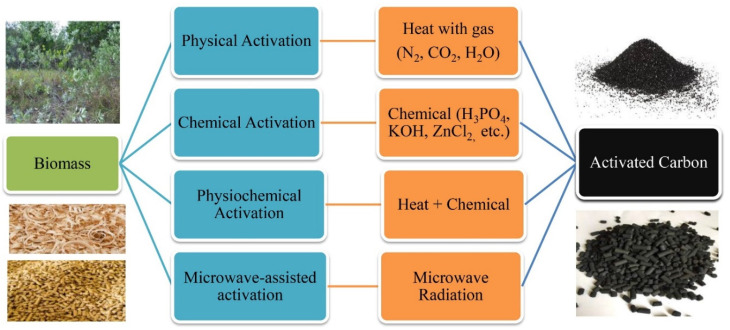
Synthesis of activated carbon by various processes and activating reagents [36].

## 2. Synthesis Methods of Activated Carbon

The use of waste biomass as a precursor of porous ACs has gained great attention worldwide. It is due to its bio-waste abundancy and low cost, and it has proved to be the best alternative to conventional raw materials such as petroleum residues, coal, peat, and lignite which are expensive and the majority are non-renewable [37]. There are two main methods in practice for synthesizing carbon, namely pyrolysis carbonization and hydrothermal carbonization [38]. In general practice, hard bio-waste materials are carbonized using pyrolysis carbonization, whereas soft bio-based materials are carbonized using hydrothermal carbonization. The activated carbon can be prepared by maintaining two basic steps; i) carbonization and ii) activation. Figure 2 depicts the primary activation process for the preparation of activated carbon from biomass.

### 2.1. Pyrolysis Carbonization

**Figure 3:**
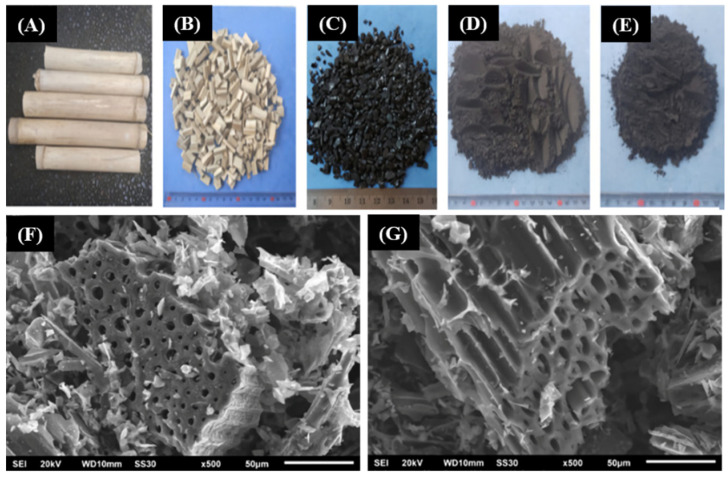
Illustration of formation of ACs using pyrolysis carbonization followed by chemical activation ((**A**) tabah bamboo; (**B**) pieces of tabah bamboo; (**C**) char; (**D**) powdered char; and (**E**) activated carbon) and SEM images obtained from bamboo (**F**,**G**) [43]. Reprinted with permission from *Surfaces and Interfaces*, Copyright Elsevier 2019.

Carbonization is the oldest process of altering biomass into carbon material for the service of humankind. It requires a relatively high temperature and is a slow and long process, and it converts organic substances into carbon or a carbon-containing residue (biochar) through pyrolysis of raw material in a furnace under an inert gas atmosphere by removing volatile, non-carbon species such as nitrogen, oxygen, and hydrogen, intensifying the carbon content [39]. During the degasification process, narrow pore structures of precursors start to develop followed by the removal of lingering substances formed when rising the temperature. Moreover, sometimes this accumulation may be the cause of collision of some lingering substances and collapse the walls of pores resulting in hydro cracking and carbon deposition [40]. In this process, temperature has the most remarkable effect along with the heating rate, the presence of an inert atmosphere and its rate, and finally, the process duration. Normally, a carbonization temperature higher than 600 °C results in a reduced yield of char and an increased liquid and gas release rate [41]. A higher temperature will also increase ash and fixed-carbon content and lower the amount of volatile matter. Thus, high temperatures result in better-quality char but a decreased yield. The decrease in the yield is thought to be caused by the primary decomposition (de-volatilization) of biomass at a high temperature and the secondary decomposition (cracking) of biochar residue. Consequently, higher temperature produces a better quality of biochar [42]. Undoubtedly, pyrolysis carbonization is the best method to produce large amounts of porous carbon products with excellent properties. Currently, this process, followed by chemical activation, is widely used to obtain bio-based hierarchical porous ACs for commercial production. The formation of well-ordered porous ACs employing this process is shown in Figure 3 where tabah bamboo is used as a carbon precursor [43]. SEM images (Figure 3F,G clearly show that the macro-, meso-, and micropores of ACs are well ordered.

### 2.2. Hydrothermal Carbonization

**Figure 4:**
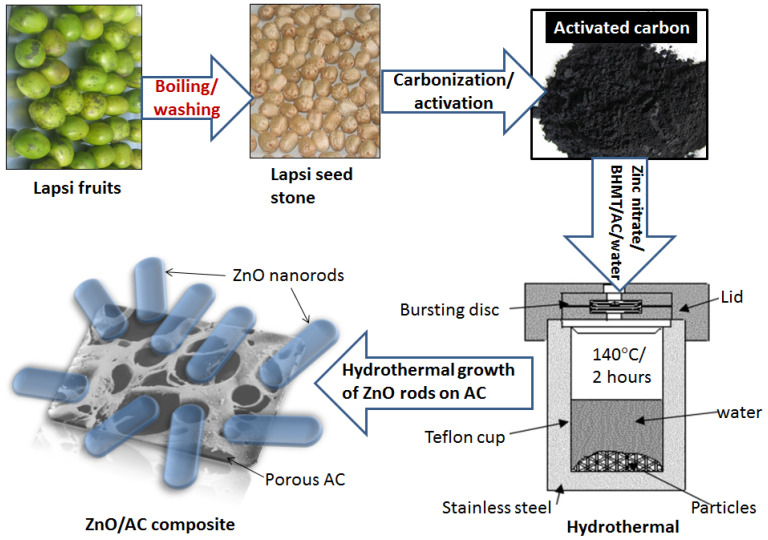
Schematic diagram showing the use of hydrothermal process for the preparation of ZnO/AC composite [35].

Hydrothermal carbonization is an alternative auspicious thermochemical process that can transmute biomass into ACs economically and environmentally friendly. Hydrothermal carbonization embraces heating raw material disseminated in an aqueous solution and autoclaving it at temperatures between 150 and 350 °C for about 2–24 h at saturated pressure (Figure 4). During this step, water-soluble components and a carbon-rich hydrophilic solid called ‘hydro char’ are formed [17,44]. The hydrothermal carbonization process has limited perquisites in terms of preparation and treatment of biomass, mainly no pre-drying needed for wet waste as in other thermal treatment processes which makes it an economically attractive alternative. The final product formation is pendent on different process parameters such as reaction time, nature of pressure, and temperature [45]. This process is led by dehydration and decarboxylation reactions which are exothermic and render the process self-subsistent after activation. At subcritical conditions, biomass is first converted into monomers by hydrolysis and then into soluble organics by dehydration and fragmentation. A reduction in pH catalyzes the initial hydrolysis step (acid catalysis) [46], and the solution concentration increases due to polymerization and condensation of material and hence nucleation, and thus the growth takes place. The chemical structure of hydro char is almost similar to that of natural coal (core–shell structure with a hydrophobic core and a stabilizing hydrophilic shell) containing oxygen functional groups such as hydroxyl, phenol, carbonyl, or carboxyl which makes it a good adsorbent. The main superiority of this process over other thermochemical conversion technologies is its potentiality to convert the wet feed stock into a solid carbonaceous product (hydro char) with high yields without exposing it to dehydration and drying [47].

Soft bio-based materials can be easily converted into biochar by this process. Thus obtained biochar can be activated by different activating agents using a tubular furnace for its surface modification. Recently, Zhao et al. reported the fabrication of ACs from tobacco rods (soft bio-waste) which produce a hierarchical porous structure with the coexistence of micro-, meso-, and macropores [48]. Moreover, the obtained ACs have numerous oxygen and nitrogen-based functional groups with excellent electrical conductivity. The hydrothermal process is considered as a simple and facile technique for the fabrication of novel materials. The process can be used for doping different metal or metal oxides on the surface of ACs. A three-step process, i.e., pyrolysis carbonization, chemical activation, and hydrothermal treatment, can be used to obtain an effective photocatalyst from bio-waste materials. Recently, our research group reported the fabrication of ZnO-rod-decorated ACs using the hydrothermal process [35]. In this work, hard bio-waste material (Lapsi seed stone) was used for pyrolysis carbonization with phosphoric acid activation followed by hydrothermal treatment with a ZnO precursor as shown in Figure 4. Phosphoric acid activation can provide the functionalities on the surface of ACs which can act as a nucleation site for the growth of ZnO nanorods on the surface of porous ACs during hydrothermal treatment. The stable incorporation of ZnO nanorods into ACs prevents the loss of the photocatalyst during recovery which can be effectively used in water treatment.

## 3. Modification Methods of Activated Carbon

Surface modification of ACs can develop proper physicochemical properties suitable for their application in environmental remediation. It is the action of tailoring the surface of ACs by adjusting the physical, chemical, or biological characteristics on their surface to produce desired physical and chemical properties [49]. Different activation methods with various activating agents can introduce unique physicochemical properties to the ACs. Synthesis of ACs by various activating reagents is presented in Figure 1, and their detailed discussion is provided in the following sections.

### 3.1. Acid Modification

It is a typical wet oxidation process applied for the fabrication of ACs from biomass. During acid oxidation, the porous carbon surfaces can eliminate mineral components and boost the surface’s hydrophilic character [24]. Strong mineral acids and powerful oxidants such as HNO_3_, H_2_SO_4_, HCl, H_3_PO_4_, H_2_O_2_, and HClO are applied for this modification [50]. Nitric acid and sulfuric acid are prominent, whereas weak organic acids are seldom used because of their weak ionization ability. Acidification of ACs enhances their acidic behavior and their surface hydrophilicity by reducing the minerals present on them. Such adsorbent materials with an acidic surface can introduce oxygen-containing functional groups such as hydroxyl, carbonyl, carboxyl, quinone, lactone, and carboxylic anhydride, etc. The presence of such functional groups on the outer surfaces or margins of the basal plane on ACs can play a notable role in adapting the material’s chemical nature [51].

An acid-treated AC can generate a positive charge on its surface, enhancing the adsorption of metal cations due to its ability to form metal complexes with anionic acid groups. Different research groups have adopted this basic principle for the adsorption of heavy metals using acid-activated carbon with various precursors of AC (derived from either coal-based materials or from biomass). Huang et al. recently reported that nitric acid is an effective activating agent for lignite which can be effectively used for the adsorption of Pb [52]. Increasing the amount of polar oxygen-containing functional groups such as hydroxyl, carbonyl, and carboxyl and the introduction of nitro groups on the surface of activated lignite make it a perfect adsorbent for heavy metals. Such chemical moieties improve the charged characteristics, the polarity of the adsorbent surface, and the metal ion adsorption capability. Along with the nature and oxidizing strength of acid, the degassing caused by heating at a higher temperature can alter the adsorption capacity of ACs. Aggarwal et al. [53] used nitric acid, ammonium persulphate, hydrogen peroxide, and gaseous oxygen at 350 °C to oxidize granular ACs and fibrous ACs. The ACs were well outgassed at different temperatures to eliminate surface chemical structures. They found that the adsorption of Cr (III) increased with oxidation and reduced with degassing. Cr (VI) adsorption, on the other hand, reduced with oxidation and rose with degassing. This result shows that metal ions having different oxidation states may have different affinities of adsorption on the same surface. Park and Jang performed HCl treatment on carbon and reported that this treatment could increase the number of surface oxygen complexes, which enhanced the active adsorbent site and, therefore, improved the reduction rates of Cr (VI) [54]. The insertion of acidic oxygen effective groups by HNO_3_ oxidation into AC was reported by Jia and Thomas [55], and the potential of modified carbon for cadmium adsorption from water was investigated. The main surface species absorbed were carboxylic acid groups, with phenol and quinone groups added during the oxidation process significantly increasing cadmium adsorption [56].

Acid treatment can show different adsorption affinities toward different heavy metals. For example, the surface of Chemviron F400, a commercial AC, was modified by the oxidation with HNO_3_ to introduce a range of functional groups on its surface [57]. After oxidation, the exterior surface area and pore void were found to be reduced. The carbon surface, on the other hand, developed an acidic nature, with carboxylic groups dominating the surface functional groups. The acid-treated sample demonstrated poly-functionality and cation-exchange characteristics throughout a wide pH range. Nickel (II), cobalt (II), copper (II), zinc (II), and manganese (II) were removed from both carbon samples. It was discovered that the affinity order Mn^2+^ < Co^2+^ < Ni^2+^ < Cu^2+^ > Zn^2+^ corresponded to the stability of their metal complexes. Modified ACs were used to study the removal of colors in addition to metal ions. Ayan et al. [58] used origanum onites stalks in their pristine and acid-modified forms using HNO_3_ and H_3_PO_4_ solutions as adsorbents to remove Basic Red 18 and methylene blue from water. They recorded that the modified ACs increased the adsorption capacity by 56–63% for Red 18 dye and 125% for methylene blue dye. Carvajal-Bernal et al. [59] performed a surface modification of ACs with H_3_PO_4_ without subsequent heating which enhanced the adsorption rate of 2, 4-DNP. It reflects the changes in the surface chemistry of carbon associated with the interaction between H_3_PO_4_ and oxygen groups present on the surface. The modification causes the acid to be dispersed on the carbon matrix and thus facilitates the retention of 2, 4-DNP.

The adsorption capacity of acid-treated ACs may depend upon the process temperature. Pradhananga et al. **[60]** fabricated nanoporous activated carbon materials from bamboo (*Bambusa vulgaris*) cane by chemical treatment with H_3_PO_4_ at different temperatures (400, 500, and 600 °C) for removal of wool carpet dyes, Lanasyn orange and Lanasyn gray. Irrespective of the carbonization temperature, it was reported that all the ACs contained oxygenated surface functional groups such as –OH, –CO, -COOH, and lactones. They found that the bamboo-derived AC prepared at 600 °C is a favorable adsorbent for the wool carpet dyes. Shrestha et al. [61] investigated the efficiency of AC prepared from Lapsi (Choerospondias axillaris) seeds by carbonization and subsequent acid treatment with concentrated H_2_SO_4_ and conc. HNO_3_ for heavy metal adsorption. The FTIR results confirm that the chemical treatment using concentrated sulfuric acid carbonized the cellulose structure of the raw material differently than the physical treatment and further oxidized carbon with concentrated HNO_3_ considerably increased the number of functional groups. Furthermore, the FTIR spectrum of the pristine carbon was significantly different from the spectra of acid-treated carbon. They demonstrated that heavy metal ions form complexes with the surface carboxyl and lactone groups in the midst of hydrogen ion development. The sorption capacities of the carbon for removal of lead and nickel, respectively, were found to be (q_m_ for Pb = 424 mg g^−1^ and q_m_ Ni = 70 mg g^−1^ at pH = 5) in the model solution. Some current research conducted on the alternation of ACs using acid treatment is summarized in Table 2.

According to the above discussion, acidic modification of AC can produce a large number of effective moieties on the surface of carbon which has been found to improve the sorption of different pollutants due to changes in its surface chemistry. As a result, the capacity of acidic treatment is directly linked to the quantity of acidic functional groups [68].

### 3.2. Alkaline Modification

A base (alkaline) treatment of AC generates a positive charge on its surface which aids in the adsorption of different negatively charged species. The easiest way of enhancing the basic surface properties in porous carbons is the treatment of ACs at high temperatures (400–900 °C) in an inert hydrogen or ammonia atmosphere [69,70]. Alkali treatment can improve the relative content of alkali groups as well as improve the surface non-polarity. Therefore, this process can enhance the adsorption capacity of ACs for non-polar substances.

The surface of ACs can be modified by treatment with NaOH, KOH, Na_2_CO_3_, Na_2_SiO_3_, and oxides. When AC is treated with NH_3_ at 400–900 °C, it develops the basic nitrogen functionalities on the carbon surface [71,72]. Doping of nitrogen functionalities can be achieved by treating it with nitrogen precursors (such as ammonia and amines) or activating it in a nitrogen-rich environment [73,74]. The induced amide, imide, lactone, pyrrolic, and pyridinic groups usually provide the suitable property that can uplift the interplay linking adsorbents and acidic breed by dipole–dipole interchange, hydrogen, and covalent bonding. Chen et al. [75] investigated the refitting of the surface of ACs through thermal treatment in the presence of ammonia. They reported a fourfold increase of perchlorate adsorption by the AC samples prepared at a temperature of 650–700 °C.

Recent important findings on the modification of the ACs by alkaline treatment are found to be effective for adsorption applications. Zheng et al. [76] found that the effect of NaOH modification on ACs significantly reduces the oxygen-containing functional groups on the surface of ACs. The pore void and the distinct surface area are increased with alkali concentration leading to an increase in the adsorption capacity of methane. When treating AC with NaOH, Chiang et al. [77] discovered that the AC enhanced the congregation of phenolic functional sites on the surface. J. Przepiorski [78] reported the impact of the heating situation on the adsorbent through ammonia treatment and found that, at an optimal temperature of 700 °C, the adsorbent’s absorption capability toward phenol rose by 29%. P-Chlorophenol (PCP) adsorption from an aqueous solution on ACs with basic characteristics has been investigated [79]. The preparation of ACs can be carried out by two methods. In the first method, a commercially available activated carbon (CWZ22) was modified at high temperatures in an ammonia-, nitrogen-, and hydrogen-rich environment. In the second method, AC was carbonized and subsequently activated to make chars using CO_2_ and steam as activating agents. The fundamental of broiled CWZ activated carbon was primarily caused by the deletion of oxygen functions, whereas the basicity of N-polymer-based ACs was caused by the high nitrogen content (2.42–5.42 wt %). The results show that π-π interaction allying the phenol ring and the graphene layers was primarily responsible for aqueous solution adsorption on the surface of ACs. PCP uptake was shown to have a modest contribution from nitrogen-derived basic sites. Kasnejad et al. [80] introduced a new method for nitrogenating commercial AC by treating the adsorbent with heat: (i) under NH_3_ atmosphere, after pre-oxidation with HNO_3_, and (ii) without pre-oxidation, for Cu (II) adsorption. The result shows that the pre-oxidation of adsorbent increased the amount of nitrogen functional groups on the structure of the adsorbent, and the modified adsorbent showed a higher Cu (II) adsorption capacity. Similarly, Ofudje et al. [81] employed an alkaline modified coconut shaft as an adsorbent removal of Pb^2+^ from an aqueous solution. They reported that the optimum removal of Pb^2+^ was 17.6 and 22.1 mg/g by pristine and alkaline-treated biomass, respectively, at pH 4.0. The authors claimed that this improvement in the adsorption capacity of alkaline-treated AC was due to an appropriately refined feature of the material. Joshi et al. [82] evaluated the characteristics of NaOH-treated nanoporous activated carbons derived from Lapsi (*Choerospondias axillaris*) seed stone, synthesized under different conditions of activation/carbonization. FTIR data confirmed that despite the different preparation conditions used, the ACs contained common oxygen-containing surface functional groups such as –OH, –C O, COOH, and lactones. The prepared carbon with carbonization at 400 °C for 3 h had the highest surface area of around 1000 m^2^ g^−1^ and showed a high adsorption capacity of around 200 mg g^−1^ for removing methylene blue dye from an aqueous solution. Modification of the AC surface using ammonia gas is also a popular strategy to improve the adsorption capacity of ACs. Different reports show that the CO_2_ adsorption capacity of NH3-modified ACs is remarkably increased which is probably the affinity of CO_2_ molecules (acidic nature) toward the basic surface of ACs. Moreover, other pollutants are also effectively removed by NH_3_-treated ACs. Some reports of the use of gaseous ammonia to modify activated carbon and its application in adsorption are provided in Table 3.

Altogether, one can understand that under alkaline conditions, hydroxyl ion reacts with surface functional groups of ACs. This change also increases the positive charge on the surface of the ACs which are capable of intensifying the negatively charged moieties from water.

### 3.3. Impregnation

Impregnation is defined as the uniform distribution of other species (in nano/micro scale) on the surface of carbon materials [35,89]. It is a process of surface decoration of porous material with metal/metal oxide/chemicals. Dry, as well as wet, impregnation methods can be employed for this process. In the dry impregnation method, a solvent can be applied to pack the aperture of the adsorbent. However, in the wet impregnation method, an excess solvent is added after the pores are filled. Metals or polymeric compounds that do not affect pH are often used as impregnating materials. According to Henning and Schafer [90], impregnation of ACs can improve the material’s prevailing properties by increasing catalytic oxidation abilities, promoting harmony among the ACs, and enhancing the material’s adsorption capabilities of the component as a passive penetrable bearer. Hydroxides, carbonates, chromates, and nitrates are common examples of impregnating materials. It is widely accepted that impregnation with appropriate chemicals significantly enhanced the adsorption abilities of AC for eliminating hazardous chemicals such as heavy metals (arsenic, mercury, and cadmium), fluoride, and cyanide in water [91]. The impregnation of ACs with metals such as silver [92], copper [93], aluminum [94], and iron [95] has gained wide interest due to their higher adsorption capacity. Moreover, the impregnated adsorbent can also provide the advantage of stability and favorable regeneration capacity [96].

Compounds of iron have been mostly utilized in fabricating the impregnated ACs. Huang and Vane [97] remodeled the ACs with a solution of iron salt to enhance their arsenic adsorption capacity. They reported that Fe-modified AC exhibits 10 times greater removal efficiency compared to pristine ACs. The efficacy of arsenic withdrawal was allocated to surface assimilation of ferrous ions and arsenate complexes formation.

Chang et al. [98] described a novel multistep process for impregnating granular activated carbon (GAC) with ferrous chloride as a precursor for excellent removal efficiency of arsenic from wastewater. Impregnation of Fe on ACs can form nano-sized particles that existed in both amorphous and crystalline forms. Iron-permeated GACs (Fe-GACs) were served with NaOH to sustain the Fe in the GAC, and the impregnated iron was found to be extremely stable in water treatments across a wide pH range. To evaluate arsenic adsorption capacities, an isotherm test was performed using synthetic-arsenate-contaminated drinking water. When the iron concentration was less than 4.22%, the iron usage efficiency remained high (40–46 mg As/g Fe). When the iron concentration was raised to 12.13%, the iron utilization efficiency dropped to 14 mg As/g. For arsenate adsorption, Fe-GACs showed better performance in an acidic environment compared to a basic one. With a pH less than 6.0, the arsenate clearance rate was about 100 percent, but with a pH of more than 7.0, it dropped rapidly. Vitela-Rodriguez et al. [99] created a variety of ACs that were treated with iron hydro (oxide) nanoparticles and evaluated for their capacity for the adsorption of arsenic from water. The maximum arsenic adsorption capability of the modified ACs ranged from 370 to 1250 g/g. Meanwhile, if the pH of the solution was increased from 6.0 to 8.0, the arsenic adsorption magnitude decreased by 32%. In contrast, the temperature had no mastery on the adsorption of arsenic. Furthermore, the presence of competing anions such as SO_4_^2−^, Cl^−^, and F^−^ significantly reduced arsenic adsorption when groundwater was utilized in the experiment.

Monser and Adhoum [100] modified ACs by impregnating them with tetrabutyl ammonium (TBA) and sodium diethyl dithiocarbamate (SDDC) for the intake of Cr (VI), Cu (II), Zn (II), and CN^−^ ions. They found from the experimental results that TBA-modified ACs display a five times greater removal performance than pristine ACs. Similarly, SDDC-modified ACs show four times greater intake of Zn (II) and Cu (II) and two times greater adsorption of Cr (VI) than pristine ACs. It is caused by the ion exchange mechanism by the impregnated chemical species on the surface of ACs. Dastgheib et al. [74] reported the effectiveness of the elimination of iron-impregnated ACs’ dissolved organic matter (DOM) from natural water. They found that ammonia treatment of oxidized carbons at a high temperature enhances DOM absorption efficiency by up to 120%. Shah et al. [101] introduced iron on AC by wet oxidation and applied both impregnated and unimpregnated ACs for the elimination of methylene blue (MB). They reported that Fe-AC exhibits MB removal of up to 95%, higher than unimpregnated ACs in the pH range of 7–10. In addition to iron, some other metals such as Al, Mn, Ni, Ag, and Zr are also used to impregnate AC. Sonal et al. [102] used zirconium-impregnated ACs to remove a reactive dye and reported a higher adsorption capacity of 500 mg/g by the pristine ACs compared to 506 mg/g by zirconium ACs. Some chemically impregnated species on AC and their potential applications are presented in Table 4.

It can be concluded that the primary advantages of impregnated ACs involve the expansion of the catalytic characteristic and enhanced synergic effect between ACs and impregnating species. However, to ensure the feasibility of the process, more research in the field of leaching of impregnated materials on ACs should be emphasized.

### 3.4. Ozone Treatment

One way to change the carbon’s structure is by its exposure to ozone due to oxidation. The combined ozone AC treatment modifies the surface groups and chemical composition of ACs [107,108]. The single procedure of interaction of ozone and ACs may be the best alternative for the removal of toxic organic compounds [109]. Some previous studies have reported that, besides adsorption, some other phenomena such as reactions between ozone, the adsorbed organic matter, and the generated free radicals can enhance the efficiency of the system [110,111].

The influence of ozonation on the surface topography and chemical alteration of two granular ACs, F400 and AQ 40, and their capacity for the adsorption of phenol (P), P–Nitro Phenol (PNP), and P–chloro phenol (PCP) from an aqueous solution have been reported [112]. It is found that the ozone-treated carbons’ porous structure remained almost unaltered compared to the plain AC. The ozone-treated AC at either room temperature (25 °C) or 100 °C produced acidic surface oxygen groups on its surface. At 25 °C, carboxylic groups predominated, but at 100 °C, a more uniform dispersal of hydroxyl, carbonyl, carboxylic, and lactonic groups emerged. Physical adsorption was caused by dispersive interactions between the electrons of the aromatic ring and those of the carbon basal planes, whereas irreversible adsorption was caused by the decay incorporation of phenolic compounds brought about by basic surface oxygen groups (SOGs). The exposure of GAC to ozone reduced its capacity to adsorb P, PNP, and PCP at ambient temperature, but when ozone was administered at 100 °C, carbon adsorption was not inhibited, and in certain cases (P and PNP on F400), it was significantly accelerated. Another research study looked at the use of ozone with heat treatment to alter the surface chemistry of ACs [113]. Cherrystones were carbonized at 900 °C and activated in CO_2_ or steam at 850 °C to create ACs. This product was ozonized at room temperature. It was concluded that the use of ozone in combination with heat treatment can make ACs a potential candidate in water treatment. Valdes et al. [114] reported the effect of ozone modification on the surface properties of ACs. Commercial AC, namely FILTRASORB 400 (Calgon Carbon Corp.), was treated with ozone with a constant flow (76 mg of O_3_/min) at 25 °C and 1 atm for different exposure times. Its adsorption proficiency was appraised by employing methylene blue adsorption. It is reported that using ozone and ACs together in wastewater treatment could change the chemical characteristics of the surface of ACs [115]. When activated carbon is exposed to ozone gas for a relevant time, the chemical entities of the carbon surface change, resulting in acid groups such as carboxylic acid, anhydride, and lactones. The adsorptive behaviors of the original carbon are influenced by changes in the oxygenated group distribution on the surface of ACs. The ozone activity has an effect on the textural properties of carbon and enhances the adsorptive properties.

### 3.5. Plasma Treatment

Plasma treatment is becoming a prominent technology for the nano-scale surface modification of different materials. It is a source of a strong electric field, charged particles with sufficient energy, powerful oxidizing as well as reducing species, UV light, ultrasound, electrohydraulic cavitations, and shock waves [116,117,118] which are responsible for carrying out the plasma treatment. One of the most widespread processes for plasma treatment of AC is the plasma oxidation process. In this process, the ACs can be subjected to plasma in the presence of regulated air or oxygen under vacuum or atmospheric pressure. During plasma oxidation, chemical addition of oxygen takes place at the carbon surfaces as oxygen free radicals and increases the surface acidity which alters the surface chemistry of ACs significantly.

Recently, the use of plasma modification of ACs for the removal of hazardous organic compounds from wastewater has gained significant development. The treatment of ACs with oxygen plasma can be carried out to produce oxygen-containing functional groups onto the surface of ACs [119]. It is reported that oxygen functional groups such as C_6_H_5_OH and O-C = O are increased while the specific surface area is slightly decreased during a plasma treatment. Because of the high oxygen concentration and well-developed micropores, plasma treatment is very facile and economic for removing highly toxic materials.

The surface modification of ACs by low (cold) oxygen plasma for the study of adsorption of dibenzothiophene (DBT) in diesel fuel was studied by Zhang et al. [120]. They found that, owing to an improvement in the surface congregation of acidic oxygen-containing functional groups, the adsorption efficiency of the plasma-treated ACs rose by 49.1% compared to the pristine ACs. In another study, the surface modification of AC fiber was redesigned by gilding arc discharge technology for the adsorption of Acid Orange II from wastewater [121]. It was claimed that the plasma-modified AC’s adsorption efficiency increased by 20.9% compared to the original one. This is owing to improvement in the amount of surface oxygen-containing functional groups.

### 3.6. Biological Modification

Biological AC technology is a combination of degradation by efficient microbes and an AC-based adsorption process [122]. In the biological modification, bacteria are confined within the ACs and begin to accumulate under a suitable temperature and nutrients for growth [123]. It is found that biological modification has a crucial distinct outward area and a well-developed pore structure on ACs. Therefore, they can productively engross solvated oxygen and organic subject in water. In addition, the attached microorganisms convert the degradable part, carbon dioxide, waste products, and biomass prior to material employing adsorption situated on the ACs.

The biological AC system is based on the interaction among AC particles, microorganisms, contaminants, dissolved oxygen, and other pollutants present in the soluble state in wastewater. Microorganism adsorption on AC may be advantageous in various ways. For example, carbon-bed life may be extended in biologically modified ACs by converting a fraction of refractory organics to biodegradable organics. The microorganism then converts the biodegradable portion before this substance may employ adsorption situated on the ACs. Furthermore, the biofilm produced on ACs can alter its surface energy, potentially improving its adsorptivity [124].

Chen, M. et al. discovered that adding polyvinyl alcohol (PVA) to powdered AC as an entrapment agent for immobilized microorganisms accelerates the mass transfer process of the reaction system and has the efficacy of both adsorption and entrapment [125]. Gutierrez et al. [126] found that ACs served as a carrier for microbial immobilization and improved the treatment efficacy of p-phenol by around 30%. As a result, AC has a synergistic effect on microorganisms and their processing capacities, boosting their adsorption efficiency significantly.

### 3.7. Microwave Treatment

In recent years, microwave treatment has been proposed as a potential strategy for the modification of AC due to its benefits of fast temperature increase, uniform temperature distribution, and energy savings over traditional heating methods [127,128]. Microwaves supply energy to the carbon particles using dipole rotation and ionic conduction and have been long-established in the fabrication and regeneration of ACs [129]. Furthermore, microwave processing networks are also somewhat flattened, convenient, sustainable, and profitable which can improve the adsorption capacity of ACs. Liu et al. [130] prepared modified bamboo-based ACs by microwave treatment under an inert atmosphere of nitrogen. The ACs showed enhanced adsorption of methylene blue after the modification, which was attributed to the increased micropores in the modified ACs. ACs from corncob furfural residue have also been prepared by microwave irradiation using zinc chloride [131]. The impact of the ratio by weight of ZnCl_2_ and the time of soaking the solution, time of microwave radiation, and the ZnCl_2_ solution’s pH value was examined. It was reported that the best-fitted parameter for microwave-modified ACs was ZnCl_2_ to corncob furfural residue with a ratio by weight of 3.5:1, irradiation time 20 min, soaking time 12 h, and pH value of the ZnCl_2_ solution.

### 3.8. Grafting of Different Moieties

The modification of activated carbon has received much attention to increase the selective adsorptive properties of ACs. Among the several methods of modification of ACs, grafting is also one of the promising methods. Grafting of chemical agents onto activated carbon surface facilitates the binding of functional groups from the chemical agents to the porous carbon surface assisted by new bond formation caused by chemical reaction. Functionalization of carbon surface by grafting is terminated usually by its outpouring in chemical solvents furnishing functional moieties that are eventually bonded chemically to the carbon chain affording the much-needed temporary stability [132]. The leading advantage of functionalization by grafting on the surface of ACs is the gripping of functional groups by bond formation [133]. However, the disadvantage of grafting functional groups is that it is less sensitive compared to free groups due to the aversion outcome from bond formation [27]. This method is also affected by limited chemical loading accomplished when surface carbon has been utterly occupied.

## 4. Application of Bio-Based Modified Activated Carbon in Adsorption

Due to the large specific surface area, low production cost, and ease of handling and modification, bio-based ACs can be considered as ideal materials in different applications [134]. The modified ACs are applied for various applications such as gas adsorption, energy storage, and water treatment. It can be exploited in the adsorption of CO_2_, NO_x_, organic pollutants, dyes, etc. ACs are increasingly used in water purgation for the removal of pollutants and contaminants. It is reported that around 80% of the AC around the world is being used in a liquid phase or aqueous solution [135]. The adsorption of pollutants from the solution is an exchange process that mainly depends on the porous structure and surface area of the ACs [16,136]. The adsorption mechanism may be influenced by the electrostatic or non-electrostatic interaction between the solute and the carbon surface. Electrostatic interactions arise due to the ionization of electrolytes, whereas the non-electrostatic interactions are largely due to dissipation and aquaphobic interaction.

Compared to pristine ACs, surface-modified activated carbon shows better adsorption and catalytic activity. Different research groups have reported various surface-modified ACs. Recently, Joshi et al. [137] investigated the efficiency of a magnetic Fe_3_O_4_/sugarcane bagasse (*Saccharum officinarum*) AC composite for the adsorption of arsenic (III) from aqueous solutions. AC was prepared from sugarcane bagasse by treatment with H_3_PO_4_ at 400 °C. The AC was composited with Fe_3_O_4_ particles by facile one-pot hydrothermal treatment. They found that the composite could remove the arsenic from the water far more effectively than plain AC. The highest percentage of arsenic removal was found at pH 8, adsorbent dose of 1.8 g/L, and contact time of 60 min. The equilibrium experimental data showed the best fit to the Langmuir model with a maximal adsorption capacity of 6.69 mg/g.

ACs have been studied by many researchers as promising catalysts for toxic gases. Altering the pore size and surface chemistry of ACs can effectively change the catalytic oxidation of some toxic gases such as NO. Recently, You et al. reported that ACs based on hydrothermally HNO_3_-treated wood can have better catalytic activity toward NO compared to metal oxide, especially at room temperature. The adsorption of pollutants by ACs from biomass is presented in Table 5.

## 5. Concluding Remarks

A comprehensive study based on various modification techniques on AC derived from biomass to increase its adsorption ability was presented. Various techniques have been established to develop a new generation of various surface functional groups in ACs, including acid treatment, base treatment, salt treatment, ozone treatment, impregnation treatment, plasma treatment, and microwave treatment. The experimental results indicate that the acidic treatment requires uptake of metal ions while base treatment is applied mainly for the removal of anionic and organic compounds from an aqueous environment. Findings on ACs with surfactant amendments, which are designed to remove contaminants from aqueous solutions, are limited and therefore require additional exploration to impart excellent performance. The plasma technique for generating an appropriate charge on superficial carbon is an efficient method to create the desired charge on the AC surface making it beneficial for the elimination of toxic pollutants. In addition, carbon-based materials with basic surfaces to incorporate CO_2_ adsorption in order to attenuate global warming are extremely desirable. On the other hand, further investigation is required of the surfactant modification of ACs that can be tailored to eliminate pollutants from an aqueous environment to yield excellent performance as experimental findings in this field are limited. To select a modifying agent, some factors such as the charge (positive, negative, or neutral), state (solid, liquid, gaseous), size, solubility, and pH of the system are required. However, some of the major drawbacks of modification methods include the associated costs involved in the process and the leaching of hazardous chemicals employed in the alternation process into the treated water. Considering the effects of increased/decreased smell of certain contaminants, selective adsorption may be generated, and the reclamation of the adsorbents should be utilized in cyclic measurements and quietly. Considering the above, the area demands novel, sustainable, and environmentally friendly approaches for activated carbon modification. Thus, the authors suggest that existing modification procedures should be improved as these techniques enhance the chemical surfaces of ACs while limiting the degradation of textural features. It would also promote the dominance of the AC adsorption method of water/wastewater treatment and other applications.

## Figures and Tables

**Table 1:** The biochemical composition of some agriculture lignocellulosic biomasses [14].

Feedstock	Lignin (%)	Cellulose (%)	Hemicellulose (%)
Almond shell	20.4	50.7	28.9
Bamboo	21.31	26–43	15–26
Banana waste	14	13.2	14.8
Barley straw	14–14	31–34	24–29
Rice straw	18	32.1	24
Sugarcane bagasse	23–32	19–24	32–48
Tea waste	40	30.2	19.9
Walnut shell	52.3	25.6	22.7
Wheat straw	15–20	33–40	20–30
Wood	25–30	35–40	20–30

**Table 2:** Some reports on the alteration of AC with acid treatment.

Samples	Acid Used	Species Biosorbed	Ref.
Agricultural waste	H_3_PO_4_, H_2_O_2_	Cd	[62]
Activated coconut shell carbon	H_3_PO_4_	Zn(II)	[63]
Aquatic weeds	H_2_SO_4_	Cr(III), Cr(VI)	[64]
Olive mill solid residue	HCl	Phenol	[65]
Olive stone	H_2_SO_4_, HNO_3_	Pb (II)	[66]
Rice bran	HNO_3_	Cd(II), Cu(II), Pb(II), Zn(II)	[67]

**Table 3:** A review of studies regarding the use of gaseous ammonia to modify activated carbon.

Materials	Amination Temperature	Applications	Ref.
Carbon materials (biomass residues, sewage, sludge, pet, coke)	400 °C	CO_2_ adsorption	[83]
Commercial granular activated carbons	385 °C	Adsorption of aromatic compounds (aniline, nitrobenzene)	[84]
Activated carbon from sulfonated styrene-divinyl-benzene copolymer	600 °C	Adsorption of molybdenum	[85]
Carbon adsorbents from biomass residue (almond shells)	800 °C	CO_2_ adsorption	[86]
Activated carbon from pea	900 °C	Enhancement of catalytic activity of AC in oxidation reaction	[87]
Commercial activated carbon	1000 °C	CO_2_ adsorption	[88]

**Table 4:** Chemically impregnated ACs and their potential applications.

Samples	Species Impregnated	Species Removed	Ref.
Activated carbon	iodine and chlorine	gas-phase elemental mercury	[103]
Granular activated carbon	sulfur	gas-phase elemental mercury	[104]
Activated carbon	metallic silver and copper	arsenic	[105]
Activated carbon	silver and nickel	cyanide	[100]
Granular activated carbon	copper and silver	cyanide	[106]

**Table 5:** Applications of activated carbon from biomass.

Biomass Precursor	Application	Ref.
Coconut shell	Reduction of hexamine cobalt (III)	[138]
Rice husk	Adsorption of Cu	[139]
Peach stone	Adsorption of gold	[140]
Sugarcane bagasse	Decolorization of sugar	[141]
Guava peel	Removal of Congo Red dye	[142]
Oil palm shell	Methane adsorption	[143]
Ground nut shell	Adsorption of malachite green	[144]
Almond husk	Adsorption of Ni (II) from aqueous solution	[145]
Nut shell	Methylene blue adsorption	[146]
Bamboo	Adsorption of methylene blue	[147]
